# Case report: Formation and recurrence of inflammatory pseudotumor after metal-on-metal hip arthroplasty

**DOI:** 10.3389/fmed.2024.1422230

**Published:** 2024-07-11

**Authors:** Meipeng Min, Chenyang Xing, Peili Xu, Xincheng Wei, Lei Fan

**Affiliations:** Department of Orthopedics, The Second Affiliated Hospital of Nanjing Medical University, Nanjing, China

**Keywords:** metal-on-metal (MoM) bearings, inflammatory pseudotumor, prosthesis revision, total hip arthroplasty, adverse reaction to metal debris

## Abstract

The metal-on-metal (MoM) artificial hip joint is a prosthesis used in early hip arthroplasty, particularly for hip resurfacing and total hip arthroplasty. However, abrasion and corrosion of MoM bearings result in the production of metal ions, such as cobalt and chromium, thereby inducing several complications such as inflammatory pseudotumor, aseptic inflammation, and allergy to metal ions (delayed type IV hypersensitivity). In this case report, we present a patient who was hospitalized for recurrence of a mass in the right inguinal area. In 2010, the patient underwent right MoM total hip arthroplasty for right femoral head necrosis and exhibited a good postoperative recovery. In 2019, the patient experienced pain in the right hip with activity limitation without any evident triggers, and a palpable mass was observed in the right inguinal area. A large periprosthetic mass was resected under general anesthesia, and the patient recovered well after the operation. Based on post-surgery imaging and pathological examinations, the mass was diagnosed as a periprosthetic inflammatory pseudotumor. In 2021, the inflammatory pseudotumor recurred at the same site. He then underwent right total hip revision surgery under epidural anesthesia and recovered well after surgery. No recurrence was noted at moderate follow-up. The incidence of inflammatory pseudotumors is high in MoM hip arthroplasty. Early revision is necessary in patients who meet the indications for revision, while regular postoperative follow-up is crucial.

## Introduction

Although metal-on-metal (MOM) hip implants have now been discontinued, the proportion of MoM hip arthroplasty was high in early-stage joint replacements. It was the preferred choice for young patients and patients with high activity levels because it was associated with a lower incidence of dislocation, increased joint load bearing, and the avoidance of osteolysis caused by polymeric materials such as polyethylene (1). However, MoM hip arthroplasty has recently been found to be associated with varying degrees of periarticular inflammatory pseudotumors. These pseudotumors manifest as cystic or solid soft tissue masses composed of inflammatory cells from necrotic tissue that are connected to the hip joint (2). On clinical examination, the patients may present with a palpable mass on the anterior or lateral hip surface, or even in the iliac fossa. Pain predominantly occurs in the inguinal region and occasionally radiates to the greater trochanter and lower extremities. In patients with pseudotumors, severe pain may cause them to adopt a limping gait. Over time, the patients may develop hip instability (subluxation, dislocation). Some patients may experience other symptoms such as stiffness in the lower extremities, decreased range of motion, and weakness of the abductor muscles (3). These sterile masses in the tissue surrounding the prosthesis are clinically known as inflammatory pseudotumors and are believed to result from an adverse reaction to metal ions released during the wear and tear of metal-bearing surfaces. Soft tissue inflammatory reactions in response to metal debris are collectively called adverse reactions to metal debris (ARMD) and include inflammatory pseudotumors, aseptic lymphocytic vasculitis-associated lesions (ALVALs), and metallosis (4).

In 27–32% of patients with MoM hip arthroplasty, asymptomatic pseudotumors can be detected on ultrasound and MRI scans (5, 6). Many regulatory agencies have recommended that the initial screening test be an ultrasound or metal artifact reduction sequence (MARS) MRI. Ultrasound is a cost-effective and readily available modality that is less affected by the presence of adjacent metal prostheses, with the key limitation being its user dependency (7). Because of its high specificity and sensitivity in detecting these responses and its versatility in assisting with preoperative planning and longitudinal comparisons, MARS MRI has been recommended as a first-line modality for evaluating the soft tissue surrounding the prosthesis in patients with MoM implants (8). A pseudotumor can be definitively diagnosed based on the pathological tissue obtained during revision surgery.

During a 7-year follow-up of 1,419 patients with MoM hip resurfacing, the incidence of pseudotumors was as high as 3.4%, and the incidence of asymptomatic pseudotumors has been estimated to be 4% (9). In a cross-sectional study of 148 hip joints from 111 patients, pseudotumors were present in 13 of 30 (43%) MoM THAs, 13 of 47 (27%) MoM RHAs, and 29 of 71 (41%) metal-on-polyethylene (MoP) THAs, which shows a statistically similar prevalence (10). However, pseudotumors in ceramic-on-ceramic (CoC) bearings and ceramic-on-polyethylene (CoP) bearings are rarely reported. Patients with inflammatory pseudotumors usually have poor clinical outcomes, with most of them requiring revision surgery. This severely affects the longevity of the prosthesis and the patient’s joint function (3).

Studies on revision surgery for recurrent inflammatory pseudotumors are rare. This report contributes to the research by presenting a case of successful revision surgery in a patient experiencing recurrence following inflammatory pseudotumor resection.

## Case report

A 64-year-old male patient was hospitalized complaining of recurrent right groin swelling and right hip pain with movement for 6 months. He had a history of hypertension and coronary artery disease. The patient underwent a right MoM total hip arthroplasty (ASR XL Acetabular System, Johnson & Johnson Medical Ltd., Shanghai, China) in our hospital in 2010 for right femoral head necrosis, a left CoP total hip arthroplasty (CORAIL Total Hip System, Johnson & Johnson Medical Ltd., Shanghai, China) in our hospital in 2017 for a traumatic left femoral neck fracture, and resection of a large periprosthetic mass in 2019. This mass was diagnosed as an inflammatory pseudotumor. The pseudotumor recurred at the same site and he underwent revision surgery (CORAIL Revision Hip System, Johnson & Johnson Medical Ltd., Shanghai, China) in 2021. As of 2024, the pseudotumor had not recurred at the 3-year follow-up.

### Examination

At physical examination, the spine had a good physiological curvature with no deformity. The patient reported no compression or percussion pain in the spinous processes. The patient experienced pain in the right hip with a limited range of motion. The right lower limb was swollen, and a large mass was palpated from the right lower abdomen to the right inguinal area without obvious compression pain. The remaining limbs exhibited no joint redness, joint ankylosis, muscle tenderness, muscle atrophy, or varicose veins in the lower limbs. Peripheral circulation, limb sensation, and muscle tone were good. The Harris Hip Score (HHS) was 87 points (The observation indices primarily included four aspects: pain, function, deformity, and joint mobility. Excellent: 90–100; good: 80–89; acceptable: 70–79; and poor: ≤69) (11).

During the laboratory examination, the glucose level decreased to 1.54 mmol/L, whereas the levels of lactate dehydrogenase, adenosine deaminase, chlorine, protein, and the erythrocyte sedimentation rate increased to 2226.0 and 67 U/L, 100.8 mmol/L, 39,357.00 mg/L, and 28 mm/h, respectively. The blood cobalt (Co) and chromium (Cr) ion concentrations determined by inductively coupled plasma/mass spectrometer (ICP/MS) techniques were 12.5 and 9.2 μg/L, respectively, on 12 April 2021.

**Figure 1:**
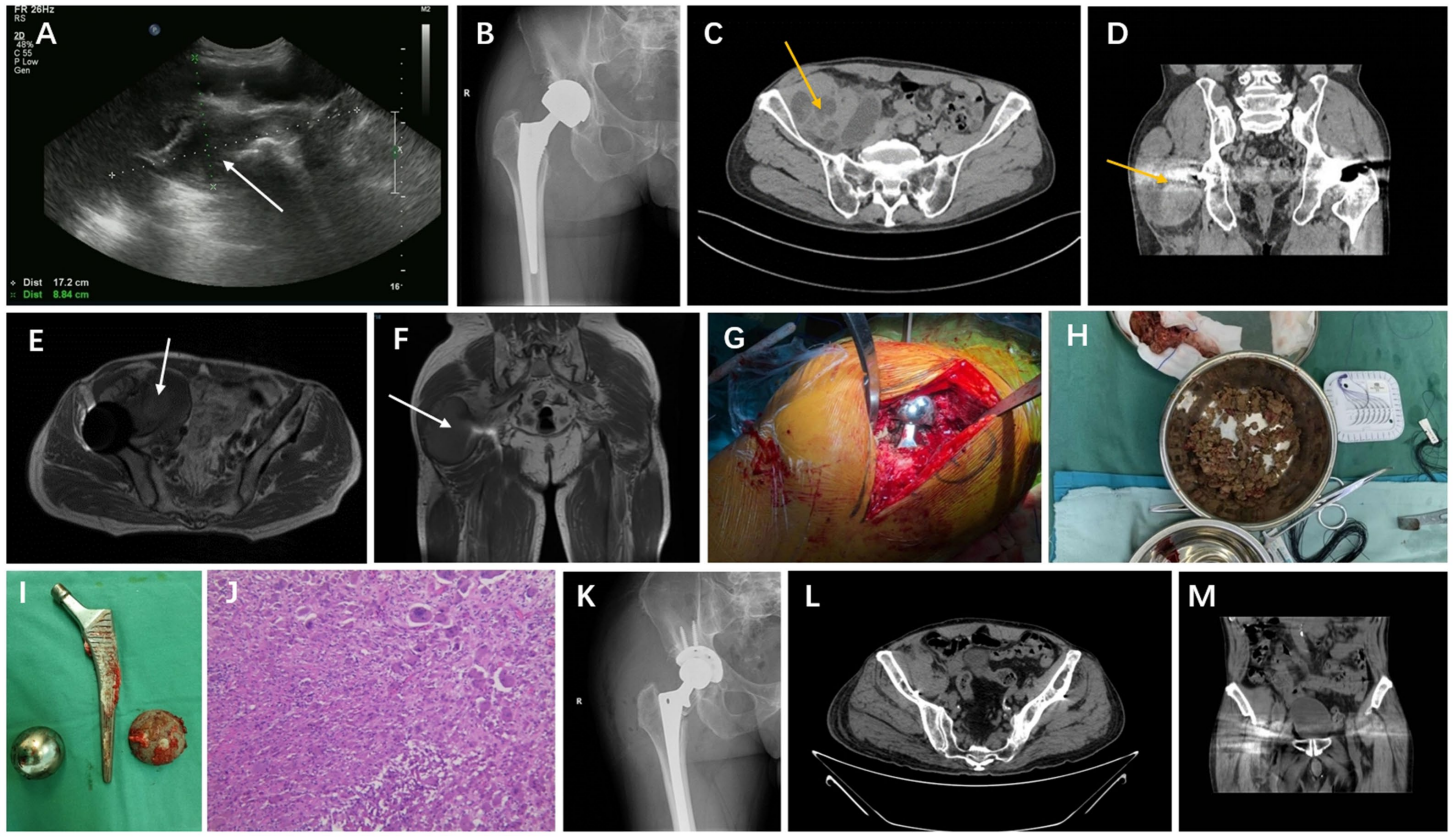
A 64-year-old male patient underwent revision surgery for recurrent inflammatory pseudotumor after MOM hip arthroplasty. **(A–F)** Preoperative ultrasonography, X-ray of the hip, CT horizontal plane, CT coronal plane, MRI horizontal plane, and MRI coronal plane; **(G)** intraoperative exposure of the prosthesis; **(H)** intraoperatively resected inflammatory pseudotumor; **(I)** removed MOM prosthesis; **(J)** pathological examination of resected material, H&E, magnification, 200X; **(K–M)** review of the X-ray, CT horizontal plane, and CT coronal plane after revision surgery.

On ultrasonography [Diagnostic Ultrasound System and Transducer, Philips Healthcare (Suzhou) Co. Ltd., Suzhou, China], a solid mass measuring 17.2 cm × 8.8 cm was observed extending from the right lower abdomen to the right groin and around the right hip (Figure 1A). It was predominantly cystic, with clear borders and irregular margins. X-ray (DigitalDiagnost DR, Philips Healthcare Co. Ltd., Suzhou, China) revealed changes following right artificial hip arthroplasty (Figure 1B). Computed tomography (CT) (SOMATOM Definition AS, Siemens Shanghai Medical Equipment Ltd., Shanghai, China) showed a mass in the right iliac fossa and right hip perimuscular space, changes following bilateral hip arthroplasty, and degenerative changes in the bilateral hip joints (Figures 1C,D). On magnetic resonance imaging (MRI) (MAGNETOM Avanto 1.5 T, Siemens Shanghai Medical Equipment Ltd., Shanghai, China), changes were observed following bilateral hip arthroplasty, and large abnormal signals were noted around the right hip joint (Figures 1E,F). An abscess was formed.

### Final diagnosis

A periprosthetic, inflammatory pseudotumor was noted after right hip arthroplasty.

### Treatment

Following successful epidural anesthesia, the patient underwent surgery in which a posterior lateral approach was used to expose and incise the joint capsule. The incision was made by cutting through the skin, subcutaneous tissue, and fascia one layer at a time. During the operation, the prosthesis was in good condition (Figure 1G), with no signs of loosening. However, a grayish-black silt-like material and necrotic connective tissue were significantly observed around the prosthesis (Figure 1H). The dislocated hip was surgically treated to remove the femoral head and stem (Figure 1I). The acetabulum and proximal femur were exposed, followed by glenoid labrum resection. The pseudotumor and necrotic tissues were also removed and subjected to postoperative pathological examination and culture (Figure 1J). After the acetabulum was polished with a grinding file, a 55-mm metal cup (Pinnacle Revision Acetabular Cup System, Johnson & Johnson Medical Ltd., Shanghai, China), two fixation screws, and a polymer polyethylene liner were implanted. Following successful trial molding, a 12-gage revision stem (CORAIL Revision Hip Stem, Johnson & Johnson Medical Ltd., Shanghai, China) was installed, and good hip movement was noted after repositioning. Saline irrigation was performed, followed by the repair of the joint capsule. After a drain was placed, the incision was closed. Following the operation, the patient was transferred back to the ward, where he was treated for inflammation, dehydration, thrombosis prevention, pain relief, and nutritional support, including administration of cephalosporins, non-steroidal anti-inflammatory drugs, low-molecular-weight heparin, and glycerol fructose. Subsequently, postoperative X-rays of the hip joint and pelvic CT scans were repeated (Figures 1K–M).

### Outcome and follow-up

The fibrous tissue capsule wall exhibited synovial tissue hyperplasia with numerous multinucleated giant cells, foam cells, and acute and chronic inflammatory cell infiltration, along with significant coagulative necrosis.

A smear from the secretion culture was subjected to Gram staining. This stained smear revealed a small number of neutrophils with no bacteria. The culture remained sterile for 4 days.

**Figure 2:**
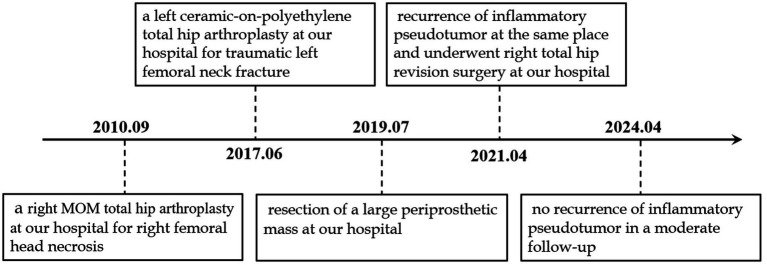
Timeline of the patient’s diagnosis with the relevant data about the treatment and follow-up.

On the second day after surgery, the drain was removed, and the patient began rehabilitation exercises, including ankle pump exercises, quadriceps exercises, hip abduction, and flexion. Repeat each set of movements 10-15 times, 2-3 times per day. On the fourth day, the patient could stand with the assistance of a walking aid and perform daily activities independently. To prevent postoperative dislocation, internal rotation and retraction of the affected limb beyond the midline and hip flexion beyond 90° were prohibited for 3 months after surgery. He was discharged from the hospital 1 week after admission, and moderate follow-up displayed no complications such as the recurrence of inflammatory pseudotumors, periprosthetic infection, or osteolysis. Figure 2 presents the timeline of the patient’s diagnosis, treatment, and follow-up.

## Discussion

Metal-on-metal interfaces have been extensively used in the early stages of clinical application in young patients with high demands on joint mobility and quality of life. These interfaces have the advantage of lowering the joint dislocation rate, increasing the joint’s range of motion, and reducing osteolysis (12). However, stable and unstable prostheses cannot avoid wear and metal corrosion after long-term use. The resulting metal debris and metal ions can trigger several immune and hypersensitivity reactions, leading to clinical problems such as inflammatory pseudotumors, heavy metal toxicity, and delayed allergic reactions, and eventual joint revision is inevitable (13). Although MoM prostheses are now rarely used because of their high complication and revision rates, 80% of prostheses are still retained in patients. An increasing number of MoM patients are expected to require revision in the future (14).

By introducing an inorganic artificial joint to replace the damaged, deformed synovial joint, total hip arthroplasty modifies the original tissue type and structure of the hip joint. However, it removes existing pathological factors while introducing new ones. In MoM joint prostheses, corrosion or abrasion of the metal material can produce metal debris and ions, as well as metal oxides such as Co, Cr, titanium, molybdenum, and nickel. This leads to increased serum and urine metal ion concentrations in the patient (15), further causing massive lymphocytic infiltration of the tissues surrounding the prosthesis, which is observed on pathological examination as tissue necrosis, deposition of wear particles with macrophage infiltration, diffuse lymphocytic infiltration, and ALVALs (a type IV hypersensitivity reaction) (16, 17). The joint capsule and periprosthetic area are first exposed to these types of wear particles and noxious irritants, such as metal ions, and aseptic inflammation also begins in these areas. When the joint fluid is dispersed to the surrounding tissues or absorbed through the lymphatic system, the wear particles and metal ions are carried, and the locally produced inflammatory factors enter the surrounding tissues or the circulatory system. Under the action of these various inflammatory factors, the joint capsule wall undergoes tissue necrosis and fibrosis becoming hardened and less elastic. Thus, the joint capsule is more likely to rupture under the effect of fluid pressure. After rupture, the fluid enters the surrounding soft tissue space and props up a new space or directly infiltrates and invades the surrounding tissues (18). A cystic mass or cystic solid mass, known as an inflammatory pseudotumor, is finally observed on MRI or ultrasound.

Several recent clinical follow-up studies have confirmed that the prosthesis failure rate after MoM total hip arthroplasty has significantly increased, with rates as high as 34% at 5 years. Laaksonen et al. (19) offered a 2010 Depuy recall of the ASR XL MoM THA product line, which demonstrated a 13% postoperative revision rate. A meta-analysis study (20) reported that the incidence of inflammatory pseudotumors is up to 6.5% in MoM hip arthroplasty. Our case patient was admitted to the hospital 9 years after MoM hip arthroplasty because of the worsening symptoms of hip pain and limited mobility. Based on imaging and pathological findings, the patient was diagnosed as having a periarticular inflammatory pseudotumor. Revision surgery was not completed until 11 years after the surgery. However, the inflammatory pseudotumor may have formed before the onset of symptoms. The inflammatory pseudotumor, osteolysis, and aseptic loosening can be clinically asymptomatic and are not criteria for prosthesis failure. However, the absence of clinical symptoms does not mean irrelevance, and these negative events will progressively lead to THA failure (21). Apart from revision, no proven effective interventions can help cease the progression of periprosthetic lesions (2). The main indications for revision are a pseudo-grade III lesion pseudotumor (solid pseudotumor) observed on imaging (22) (Grade I: cyst wall thickness of <3 mm; Grade II: cyst wall thickness of >3 mm; and Grade III: lesions were predominantly solid lesions, where the largest dimension of the solid components was greater than the diameter of the cystic components); hip symptoms exhibiting elevated whole-blood metal ion concentrations; and persistent or progressive hip symptoms. These symptoms primarily include groin pain that occasionally radiates to the greater trochanter and lower thighs, a feeling of instability, hip dysfunction, and popping sounds (23). Due to the lack of clear guidelines and the complexity of the patient’s condition, risk factors for patient management and prognosis in the clinic, such as in asymptomatic or mildly symptomatic patients with anomalies, have not been assessed accurately (19). In such cases, determining a revision surgical plan, as well as pre- and post-surgical patient management, can be challenging for surgeons.

The MoM prosthesis was comparable to other types of prostheses in terms of clinical and functional results (24). However, the release of metal debris or ions into the bloodstream because of the wear of metal bearings increases the risk of higher serum concentrations of Cr and Co ions in patients implanted with MoM prostheses (25). In the United Kingdom, the Medicines and Healthcare Products Regulatory Agency has indicated blood Cr and Co ion concentrations of >7 μg/L as a high-risk threshold for ARMD (26). In 25 patients who had MoM bearings removed, metal ion levels decreased by 90% at 12 weeks after the MoM implant was removed (27). Because of individual differences and differences in initial ion concentrations, the trend of decreasing serum metal ion concentrations varies for each patient after revision surgery. The serum concentrations of Cr and Co ions in our case patient were consistently above the threshold before the revision surgery. Moreover, metal ion concentrations exhibited no significant decrease in the short term after the bearing was replaced until 6 months later when the concentrations were reduced below the threshold. However, some studies have noted that serum metal ion concentrations and pseudotumor formation are not significantly related, which has raised concerns about the reliability of these concentrations as a suitable screening test (25, 28). Therefore, metal ion analysis should not be used alone for evaluating patients with MoM hip implants. Clinical symptoms, blood test results, and radiographic studies must all be carefully considered while predicting prosthesis failure (29).

Pseudotumor revision is significantly associated with postoperative complications, with up to 50% of patients experiencing severe complications and one-third of patients requiring further revisions (30). Women with small femoral heads, acetabular cup implant inclination >55°, and primary hip dysplasia have a worse prognosis (9). The most common postoperative complications are dislocation, pseudotumor recurrence, and aseptic loosening (31). The pseudotumor recurrence rate after revision resection may be as high as 30% (6). Various surgical, individual, and implant factors contribute to pseudotumor recurrence (19). Intraoperative incomplete debridement, or residual metal content, is the main cause of pseudotumor recurrence (32). However, in clinical practice, because the pseudotumor is connected to the joint capsule, the extent and degree of debridement need to be carefully selected to protect crucial neurovascular tissues (32). This results in an incomplete resection of the pseudotumor. In addition, extensive debridement can cause joint instability and increase the dislocation risk (33). Therefore, the surgeon must completely remove the diseased tissue and metal fragments. Otherwise, the risk of pseudotumor recurrence increases (34). In the present case, the pseudotumor recurred 2 years after the first inflammatory pseudotumor was resected. The patient was followed up every year by telephone and outpatient service after the second revision surgery to check on the status of his imaging and to learn about his postoperative joint function, and no pseudotumor recurrence has been reported so far. The frequency of follow-up needs to be individualized based on the implant risk stratification and the clinical status of the patient. According to studies (20, 35), annual follow-up is sufficient for patients with moderate- to high-risk implants. Follow-up should include history, clinical examination, functional scores, blood metal ion measurements, and ultrasound (20). If clinical concerns exist, a MARS MRI can be conducted. For patients with low-risk implants, a less intensive follow-up is required, such as annual questionnaires and 5-year clinical reviews (34, 36).

In the present case, the patient’s pseudotumor recurred 2 years after the first inflammatory pseudotumor was resected, and no pseudotumor recurrence was observed on imaging examinations since the second revision surgery. A medium follow-up revealed that the metal ion concentrations in the patient’s body were steadily remained below the high-risk threshold 6 months after the second revision surgery. Therefore, continuous postoperative imaging and laboratory examination are quite necessary to determine pseudotumor recurrence (37).

Patients who develop inflammatory pseudotumors following MoM arthroplasty and meet the indications for revision surgery should undergo early revision to prevent further osteolysis and the occurrence of pathological fractures and to reduce the complexity of revision surger (35). The surgeon needs to accurately diagnose and judge whether the patient meets the indications for revision surgery through clinical symptoms, imaging data, and laboratory data. The use of monolithic revision and ceramic-to-polyethylene interfaces in revision surgery also results in better clinical outcomes (38). Ceramic interfaces are currently popular in healthcare settings. However, ceramic-to-polyethylene and metal-to-polyethylene interfaces used in revision MoM hip arthroplasty reduced the incidence of adverse outcomes by 70 and 63%, respectively, compared with ceramic-to-ceramic interfaces (39). In addition, regular follow-up after early MoM arthroplasty and revision is crucial for detecting and understanding the size and extent of pseudotumor formation through ultrasound and MRI. To achieve a clear diagnosis, appropriate laboratory tests and pathological biopsy are necessary because distinguishing inflammatory pseudotumors from infections, tumors, and other diseases is sometimes difficult (40).

In conclusion, the occurrence of inflammatory pseudotumors in MoM hip arthroplasty is significant and is related to prosthesis-produced metal debris. Early revision in patients who meet the indications for revision is essential to avoid adverse factors, such as aseptic loosening, that affect the prognosis of revision, and regular postoperative follow-up is vital. Collecting patient and implanted prosthesis data through collaboration between healthcare providers and regulators for constructing huge data centers and developing clinical predictive models is critical for clinical decision-making.

## Data availability statement

The original contributions presented in the study are included in the article/supplementary material; further inquiries can be directed to the corresponding author.

## Ethics statement

The studies involving humans were approved by the Ethics Committee of the Second Affiliated Hospital of Nanjing Medical University. The studies were conducted in accordance with the local legislation and institutional requirements. The participants provided their written informed consent to participate in this study. Written informed consent was obtained from the individual(s) for the publication of any potentially identifiable images or data included in this article.

## Author contributions

MM: Formal analysis, Writing – original draft. CX: Formal analysis, Writing – original draft. PX: Formal analysis, Writing – review & editing. XW: Formal analysis, Writing – review & editing. LF: Formal analysis, Resources, Supervision, Writing – review & editing.
